# Sentence-level complexity in Russian: An evaluation of BERT and graph neural networks

**DOI:** 10.3389/frai.2022.1008411

**Published:** 2022-12-08

**Authors:** Vladimir Vladimirovich Ivanov

**Affiliations:** ^1^Faculty of Computer Science and Engineering, Innopolis University, Innopolis, Russia; ^2^Institute of Philology and Intercultural Communication, Kazan Federal University, Kazan, Russia

**Keywords:** sentence-level complexity, BERT, graph neural networks, sentence embeddings, text complexity, Russian language

## Abstract

**Introduction:**

Sentence-level complexity evaluation (SCE) can be formulated as assigning a given sentence a complexity score: either as a category, or a single value. SCE task can be treated as an intermediate step for text complexity prediction, text simplification, lexical complexity prediction, etc. What is more, robust prediction of a single sentence complexity needs much shorter text fragments than the ones typically required to robustly evaluate text complexity. Morphosyntactic and lexical features have proved their vital role as predictors in the state-of-the-art deep neural models for sentence categorization. However, a common issue is the interpretability of deep neural network results.

**Methods:**

This paper presents testing and comparing several approaches to predict both absolute and relative sentence complexity in Russian. The evaluation involves Russian BERT, Transformer, SVM with features from sentence embeddings, and a graph neural network. Such a comparison is done for the first time for the Russian language.

**Results and discussion:**

Pre-trained language models outperform graph neural networks, that incorporate the syntactical dependency tree of a sentence. The graph neural networks perform better than Transformer and SVM classifiers that employ sentence embeddings. Predictions of the proposed graph neural network architecture can be easily explained.

## 1. Introduction

Linguistic complexity is well-studied at various levels of linguistic units from whole texts (Collins-Thompson and Callan, 2005; Crossley et al., 2008; Heilman et al., 2008) to individual words (Shardlow et al., 2020, 2021). Sentence Complexity Evaluation (SCE) task takes an intermediate position between the text fragment level (i.e., several coherent sentences) and the level of an individual word/phrase complexity prediction. This intermediate position makes the SCE task harder to resolve. On the one hand, a sentence has fewer words than larger fragments therefore, it is not enough to collect reliable statistics (which appear to be useful for the complexity prediction of larger text portions). On the other hand, each sentence has its own structure that may affect the complexity and restrict the application of distributional semantics, which is typically used to capture complexity at a lexical level (complexity of individual words). Recent works investigate sets of features that can be used in SCE, including lexical, syntactical features from the target sentence as well as contextual features from surrounding sentences (Schumacher et al., 2016; Iavarone et al., 2021).

The SCE presents issues, especially at the levels of interpretation of the model's results and feature selection. One of the state-of-the-art approaches is deep neural networks capable to explore a wide range of features and combine them in a hierarchical and non-linear manner. For example, Vaswani et al. (2017) relate natural language processing to Transformer-based neural architectures, while Devlin et al. (2018) relate this processing to pre-trained language models (PLMs). What is more, deep neural networks have been applied in SCE before. For instance, Schicchi et al. (2020) evaluated the a long short-term memory (LSTM) model with attention mechanism in a binary classification of Italian sentences.

Brunato et al. (2018) present a detailed analysis of features that affect human perception of sentence complexity is presented. These authors study the contribution of a set of lexical, morphosyntactic, and syntactic features. The most important features are sentence length (SL), maximum dependency length in a dependency syntax tree, etc.; for sentences with the same length, the most important factors include average word length (AWL) and lexical density. A predictive model that focuses on lengths of dependencies and words tends to be more biased toward shallow representations that are likely to vary across texts, domains, etc. Such high variability can harm the robustness of modern state-of-the-art models. The main research question of this paper is following: “Can a robust SCE model ignore SL and AWL in a sentence?.” If so, “How well such a model can perform, and which features should it use?.” The secondary question is “How to interpret the SCE results given by such a model?”

This paper studies the performance of different approaches to sentence complexity prediction excluding the SL and an AWL. All the approaches incorporate deep neural representations: sentence embeddings, pure Transformer-based model, fine-tuned Russian BERT, and a graph neural network (GNN) trained on the dependency tree of a sentence. The above approaches are evaluated on both regression and classification. The derived state-of-the-art results show that successful models need to incorporate lexical and structural signals from an input sentence. The fine-tuned Russian BERT model performs the best, the GNN performs similarly, but better than the pure Transformer.

The rest of the paper is organized as follows. Section 2 discusses the current state of the art. Section 3 presents the experimental setup including the datasets and methods. Section 4 contains results of the experiments; Section 5 summarizes the paper by discussing key findings, future work, and conclusion.

## 2. Related work

As discussed above, the text complexity is a well-studied and wide area, therefore the section surveys only research works closely related to the present study. Inui and Yamamoto (2001) study the relative complexity of sentences in the readability context for deaf people. Based on a set of questionnaires, these authors collected a corpus with pairs of sentences with paraphrases. Modeling complexity was targeted on the classification of paraphrases into three levels/classes (“left,” “right,” “same”). In addition, Inui and Yamamoto developed a rule-based method and compared it to the SVM classifier trained on a set of morphosyntactic features. Later, Vajjala and Meurers (2014) evaluated an SVM classifier to predict relative complexity on a corpus of pairs of complex and simplified sentences. Maqsood et al. (2022) compare different machine learning algorithms for SCE in English dataset with seven categories.

Similarly, Schumacher et al. (2016) studied models to estimate the *relative* reading difficulty of sentences, with and without the surrounding context. The context covers at least two sentences before and after the sentence in question. Schumacher et al. bin sentences according to grade levels (e.g., a sentence from grade 1 was paired with sentences from grades 3–4, 5–6, 7–8, 9–10, 11–12). What is more, Schumacher et al. studied lexical and grammatical features (both from the target sentence and its context) to train a logistic regression classifier and Bayesian ranker. These authors show that considering the context improves predicting sentence readability. For feature selection, authors use the Random Forest Classifier revealing the most important feature in their model, “AoA” (age of acquisition of a word). The simplest model has only the AoA-based features, which allows to achieve higher score on the dataset. The study describes another interesting result claiming that 84% of sentence pairs could be answered using vocabulary features, thus justifying the high performance of the AoA features. Unfortunately, the author of this paper is not aware of any large enough lexical databases with AoA information for the Russian words.

Brunato et al. (2018) applied crowdsourcing to model human perception of single-sentence difficulty in Italian and English. These authors investigate a wide set of linguistic features and how they contribute to human perception of sentence complexity. Brunato et al. analyzed a few tens of features, such as “char_tok” (average number of characters per word) and “n_tokens” (average number of words per sentence). In their experiments, Brunato et al. show that syntactic features can play important role in defining the sentence complexity, but “char_tok” and “n_tokens” features are always in the top important features as well. What is more, Brunato et al. explicitly control the SL by binning the dataset into the sentence groups of the same length (e.g., 10, 15, 20, etc.) up to 35 tokens.

On top of the dataset collected in the previous work, Iavarone et al. (2021) presented a study of modeling sentence complexity in context. They report results for the prediction complexity in terms of MAE for SVM models and BERT models. Despite the BERT-based model demonstrating high performance, Iavarone et al. offer an interesting conclusion claiming that the BERT model does not seem to exploit syntactic features to predict sentence complexity.

Finally, deep neural networks for sentence complexity classification were proposed in Lo Bosco et al. (2021). Their model uses the TreeTagger to extract syntactic features, two LSTM layers, and a linear layer. The last layer outputs the probability of a sentence belonging to the easy or complex class. The experimental results show the increased approach effectiveness for both Italian and English, compared with several baselines such as Support Vector Machine, Gradient Boosting, and Random Forest.

## 3. Materials and methods

### 3.1. Data collection and validation

#### 3.1.1. Corpus of school textbooks

The paper uses the initial corpus of the school texts collected by Solovyev et al. (2018a). Each text in this corpus has a grade level ranging from 1 to 11. The corpus statistics are provided in Table 1. Few books in the original collection have non-integer values of grade level (e.g., 1.5 means “1st grade, advanced”). Therefore, for classification experiments, labels of such books were adjusted in the following manner: grade 1.5 transformed to 2, grade 2.5 transformed to 3, grade 4.5 transformed to 5, 10.5 and 11.5 transformed to 11 (as only one textbook had a label of 11.5). This adjustment aimed to produce fewer text categories. The result of the label transformation is shown in the “Label” column of Table 1 which can be used in both: regression and classification modes. In addition, each text was assigned a coarse category (“Category” column), which allows for evaluating models on a “3-class classification” task.

**Table 1:** Statistics on sentence length (ASL) and word length (AWL) of the initial textbooks corpus.

**File name (book)**	**Grade level**	**Label**	**Category**	**ASL**	**AWL**	**Total sentences**
year_bog_11p.txt	11.50	11	High	18.63	7.13	5,648
year_petrov_11.txt	11.00	11	High	15.65	6.71	5,029
year_guryan_11.txt	11.00	11	High	16.01	6.72	5,848
year_nik_11.txt	11.00	11	High	17.99	6.95	2,078
year_ponom_11.txt	11.00	11	High	15.71	6.65	2,190
year_plenko_11.txt	11.00	11	High	16.66	6.63	3,470
year_bog_10p.txt	10.50	11	High	19.07	6.92	5,579
year_sobol_10.txt	10.00	10	High	15.93	6.75	5,236
year_unk_10.txt	10.00	10	High	16.19	6.68	2,000
year_nik_10.txt	10.00	10	High	17.81	6.91	2,271
year_bog_10.txt	10.00	10	High	18.27	6.78	3,145
year_klimov_10.txt	10.00	10	High	17.09	6.76	3,967
year_bog_09.txt	9.00	9	High	17.88	6.68	1,710
year_nik_09.txt	9.00	9	High	16.90	6.79	2,480
year_bog_08.txt	8.00	8	Medium	17.49	6.72	2,999
year_nik_08.txt	8.00	8	Medium	15.74	6.41	1,821
year_nik_07.txt	7.00	7	Medium	15.41	6.14	1,509
year_bog_07.txt	7.00	7	Medium	15.00	6.46	1,632
year_nik_06.txt	6.00	6	Medium	15.94	6.18	1,029
year_bog_06.txt	6.00	6	Medium	15.13	5.86	985
year_nik_05.txt	5.00	5	Medium	13.11	5.57	1,566
year_vah_4pu.txt	4.50	5	Medium	15.78	5.86	1,174
year_vah_4u.txt	4.00	4	Low	13.78	6.17	1,423
year_ben_4u.txt	4.00	4	Low	12.72	6.38	604
year_gor_4u.txt	4.00	4	Low	14.75	6.56	833
year_rud_3u.txt	3.00	3	Low	14.15	5.67	1,319
year_vah_2pu.txt	2.50	3	Low	11.13	5.75	1,005
year_uch_2pu.txt	2.00	2	Low	14.14	5.73	1,559
year_uch_2u.txt	2.00	2	Low	12.00	6.05	1,621
year_vah_2u.txt	2.00	2	Low	11.10	5.73	1,100
yead_rud_2u.txt	2.00	2	Low	13.40	5.44	619
year_vah_1pu.txt	1.50	2	Low	11.22	5.69	292
year_rag_1u.txt	1.00	1	Low	8.76	5.95	74
year_rog_1u.txt	1.00	1	Low	10.33	5.95	468
year_rud_1u.txt	1.00	1	Low	12.20	5.17	495
year_lut_1u.txt	1.00	1	Low	9.74	6.36	390
year_kur_1u.txt	1.00	1	Low	9.86	6.15	200
year_vah_1u.txt	1.00	1	Low	11.17	5.38	139

As one can observe, the average sentence length (ASL) and AWL are reliable predictors of grade level. Indeed, the dataset demonstrates the Pearson's correlation coefficient between “Grade Level” and ASL of 0.91. The “Grade Level” and AWL also strongly correlate with each other (0.86). The values of the Pearson's correlation are calculated at the level of individual documents. Obviously, complex sentences are more likely to be present in books with higher grade levels. This fact is important because such a high correlation allows for transferring labels from a textbook to the sentences extracted from the textbook.

#### 3.1.2. Dataset with sentence-level annotations

**Figure 1:**
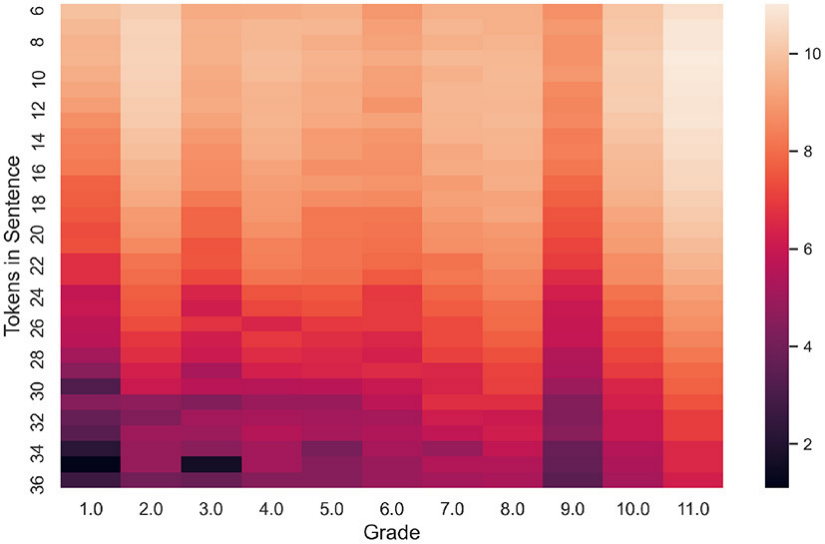
The heatmap shows the number of sentences having label “Grade” and sentence length between 6 and 36 tokens.

All documents from the initial corpus were tokenized and divided into sentences. A complexity label for each sentence was propagated from the document level to the sentence level: a textbook grade is assigned to each sentence from the corresponding text. For each sentence, the following two parameters were calculated: SL in tokens and average symbols per word in the sentence (AWL). The result of this first step is a collection of 92,536 sentences along with their complexity labels (*Grade*), SL, and AWL. The SL and AWL features will be used in baseline models. The books from higher grades are typically longer. What is more, they have more sentences and lead to an imbalanced dataset. Quite few sentences are longer than 35 tokens (Figure 1). Long sentences (longer than 35 tokens) as well as sentences shorter than six tokens were removed from the collection. After this operation, the total number of sentences in the dataset becomes 75,507.

Two directions of the present study are related to modeling of (i) single sentence complexity, and (ii) the relative complexity for a sentence pair. The first direction corresponds to the question: “How complex is a given sentence?.” The second direction aims at the question: “Which given sentence in a pair is more complex?.” A naive answer to both questions faces the same problem discussed above: the answer will be biased toward longer sentences and sentences with higher AWL values. However, many sentences have similar SL and AWL values, but come from different grade levels. In such cases, the naive approach is less applicable: one needs to collect more statistics and use other features. Therefore, ignoring the SL and AWL factors leads to a less biased sentence complexity prediction model.

Study in the first direction is denoted as “1-sentence” (or “1-s”), and the second direction aimed at assessing relative complexity is referred as “2-sentence” (or “2-s”). The construction and validation of the “2-s” dataset are presented in subsection 3.1.3 below.

#### 3.1.3. Dataset with pairs of sentences

The dataset of sentence pairs was constructed in the following way. Given two books, all possible pairs of sentences were taken from one of the two different textbooks. This set of sentence pairs was filtered according to the following criteria: (i) remove pairs with significantly different SLs, (ii) remove all pairs with significantly different values of AWL. More precisely, only pairs with the exact matching values of SL and having values of AWL deviating by not more than 0.01 from each other remained in the dataset. This dataset is significantly smaller than the set of all pairs. What is more, SL and AWL features become useless for predicting which sentence of the pair is more complex.

**Figure 2:**
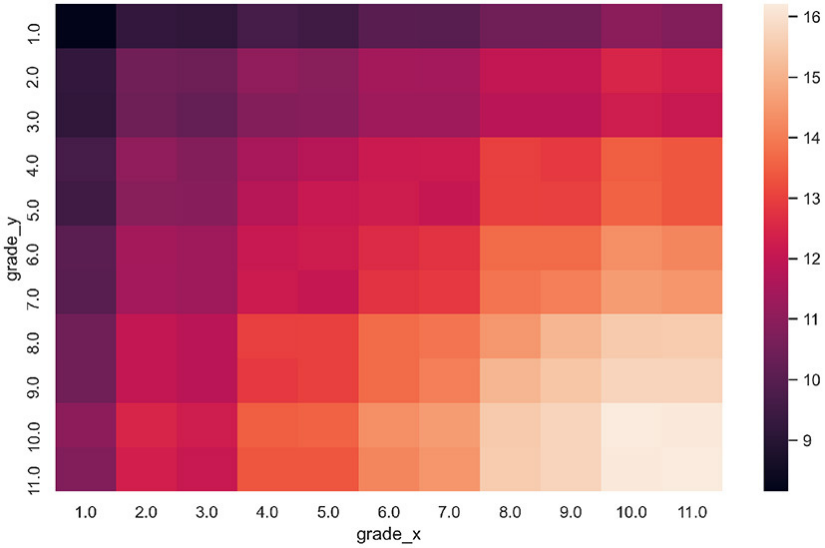
The heatmap shows the number of sentence pairs (*x, y*) where sentence *x* has label “grade_x” and sentence *y* has label “grade_y” (in logarithmic scale).

The derived 2-s dataset contains over 6 million sentence pairs. Figure 2 presents the distribution of the number of samples with respect to grade levels of sentences in each pair. This pattern is expected since the SL should gradually increase with the grade level assigned to a sentence. Moreover, Figure 2 shows distribution of number of samples on a logarithmic scale. The figure also shows the imbalance of the 2-s dataset, i.e., the higher the grade levels, the more samples are derived.

Obviously, the “Grade” of the first sentence in each pair can be equal, greater, or less than “Grade” of the second sentence. In the case of inequality, this information can be represented as a binary label. Taking into account equality allows for formulating a three-way classification task. Finally, the difference between the two grade levels shows how far the grade levels of the textbooks (sources of the sentences in the pair) are from each other. This difference can be used as a target label for regression.

Labels propagated from the textbook grades to the level of individual sentences can be noisy. For example, a simple sentence can still appear in a textbook with a high grade. Therefore, the dataset should be validated. Section 3.1.4 below presents the approach for validation of the 2-s dataset.

#### 3.1.4. Validation of the dataset

The collected dataset has a specific characteristic that school textbooks usually contain simple sentences. Typical native speakers will perceive most of the sentences as easy to comprehend. Thus, direct validation of both datasets most probably will be not fair since manual assessment of a sentence from the 1-s dataset can be biased (most assessors will assign lower grades to sentences, even to those coming from the textbooks with higher grades). Another issue with the 1-s dataset is the SL that will affect the assessment. Obviously, the ASL increases when the grade level of a book grows, but assessors tend to assign higher complexity scores to longer sentences as well. Therefore, this factor cannot be isolated completely.

To overcome these issues, a two-step approach was applied to validate data. In the first step, a random portion of all sentence pairs from the 2-s dataset was used to train a binary classifier that predicts whether one sentence in a pair is more complex than the other one. This classifier is based on the Support Vector Machines algorithm and uses sentence embeddings as input features^1^. The performance depends on *margin* between the sentences in a pair. Here, the margin is a minimum value of the absolute difference in grade levels of the sentences. This dependency, demonstrates that if a margin is large enough, then the accuracy of the SVM classifier can be almost 99% (see Table 2).

**Table 2:** The accuracy of a binary SVM classifier for pairs of sentences depends on the difference between grade levels (margin).

**Margin**	**SVM accuracy**	**No. of document pairs**	**No. of sentence pairs**
1	0.774	1,276	4,960,064
2	0.859	1,050	3,379,160
**3**	**0.884**	**904**	**2,734,835**
4	0.930	764	2,154,622
5	0.957	644	1,763,470
6	0.970	532	1,456,289
7	0.971	422	1,106,292
8	0.979	316	771,637
9	0.990	214	455,475

If the margin equals one, the classifier correctly predicts labels for more than 75% of the sentence pairs from a test set. The remaining 25% of sentence pairs are “hard” samples because the SVM classifier fails to predict a binary label correctly. Those pairs are not only hard for the classifier but can also be hard samples for human assessors. To test this assumption, a set of hard samples (1,526 sentence pairs) was transmitted to a crowdsourcing platform (Yandex Toloka) for manual annotation. In Toloka, each sentence pair was annotated by at least seven assessors and with one of four possible labels (“first,” “second,” “same,” “unknown”):

- “first” means that the first sentence is more complex than the second;
- “second” means that the first sentence is less complex than the second
- “same” means that both sentences have similar complexity;
- “unknown” means that complexity cannot be assessed (e.g., due to an error, typo, etc.).

Human judgments were compared to original labels from the 2-s dataset. The experiment was applied for different margin values, 1, 3, and 9. The larger the margin between sentences, the higher the agreement between manual assessments and labels from the 2-s dataset. The agreement for the margin < 3 was too weak while setting up margin value to 9 produces too “simple” dataset (see the last row of the Table 2). Thus, we set the margin to three and eliminate both the SL and word length factors from further evaluation, and construct a dataset that can be used to model perceived complexity at the sentence level.

The final validation step was the following. For a randomly sampled 50 sentence pairs, an expert was asked to manually assess their complexity. The resulting confusion matrix between the expert's labels and labels derived from Toloka is presented in Table 3; it shows moderate agreement between the expert and assessors.

**Table 3:** Confusion matrix between a human expert's labels and aggregated crowdsourcing labels.

**Expert's label**	**First**	**Same**	**Second**	**Unknown**
First	10	5	5	0
Same	8	4	2	2
Second	1	3	9	1

In all further experiments and evaluations, the margin parameter was fixed (margin = 3, the highlighted line in Table 2). For smaller margin values, the 2-s dataset labels are less reliable, while for higher values of the margin sentences in each pair are much easier to distinguish. A sentence in each pair at least three grade levels apart from the other sentence in the same pair. A similar approach was used to bin the 1-s dataset's labels into three categories: *low* (1–4 grades), *medium* (5–8 grades), and *high* (9–11 grades) which is presented in Table 1.

#### 3.1.5. Tasks for sentence-level complexity prediction

This subsection summarizes datasets and experimental settings to evaluate complexity prediction for the current study. In general, complexity prediction can be formulated in two modes: regression and classification. Table 4 shows five combinations of experimental settings that can be used depending on the dataset. Regression and binary classification can be formulated with the 2-s dataset, while the dataset with single sentences allows for three tasks: regression, 3-class classification, and 11-class classification.

**Table 4:** Setup of five tasks used for model evaluation in the study: two regression tasks, one binary classification, and two multiclass classification tasks.

**Dataset**	**Regression**	**Binary classification**	**Multiclass classification**
1-s Dataset	Grade level (value)	–	11 or 3 Categories
2-s Dataset	Difference between grade levels	Complex/Simple sentence	–

### 3.2. Modeling sentence-level complexity

Recent works evaluated several lexical and syntactical features for modeling sentence-level complexity. This paper focuses on neural architectures that are capable to extract features from input data and solve the task in an end-to-end manner. The models applied in the evaluation include baseline models (linear regression and SVM classifier), a transformer encoder with 1-layer of self-attention, a pre-trained BERT model (DeepPavlov's RuBERT from Kuratov and Arkhipov, 2019) and GNN. The models were tested on the five tasks presented in the previous section using accuracy, precision, recall, and F1-macro score for classification tasks; as well as *R*^2^, mean squared error (MSE), and mean absolute error (MAE) for regression. The performance of all models was evaluated using the same dataset split, i.e., 90% for training and 10% for testing^2^.

Transformer-based deep neural network architectures are well-known for their effectiveness in different natural language processing problems, including text classification, machine translation, and text generation (Vaswani et al., 2017). Bidirectional encoder representations from transformers (BERT, Devlin et al., 2018) are derived as a result of semi-supervised pre-training on a large corpus, stored as a set of neural network weights (usually, hundreds of millions or more). These representations can be fine-tuned to solve a down-stream task, such as SCE. Graph neural networks are less commonly applied to texts.

#### 3.2.1. Fine-tuning a pre-trained BERT

**Figure 3:**
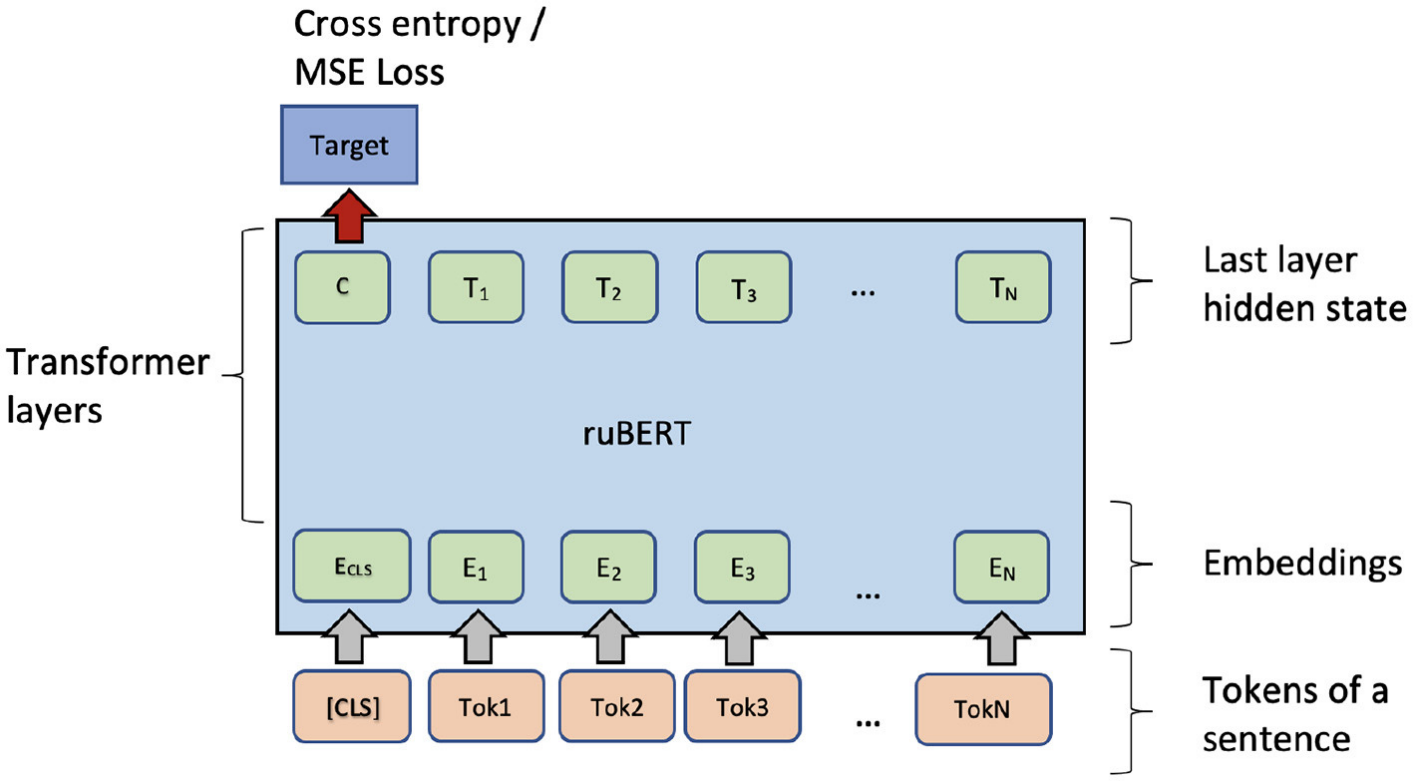
Fine-tuning of RuBERT for single sentence complexity prediction.

This study evaluates a state-of-the-art pre-trained *RuBERT* model. This model can be applied in all the settings defined in Table 4. Fine-tuning on the 1-s dataset only needs encoding the input sentence and correctly defining a loss function for the output (CLS) token and tune training hyper-parameters. Note, that the model does not explicitly depend on the input SL; however, the model can use a SL implicitly. The architecture is presented in Figure 3.

#### 3.2.2. SVM model with BERT sentence encoder

**Figure 4:**
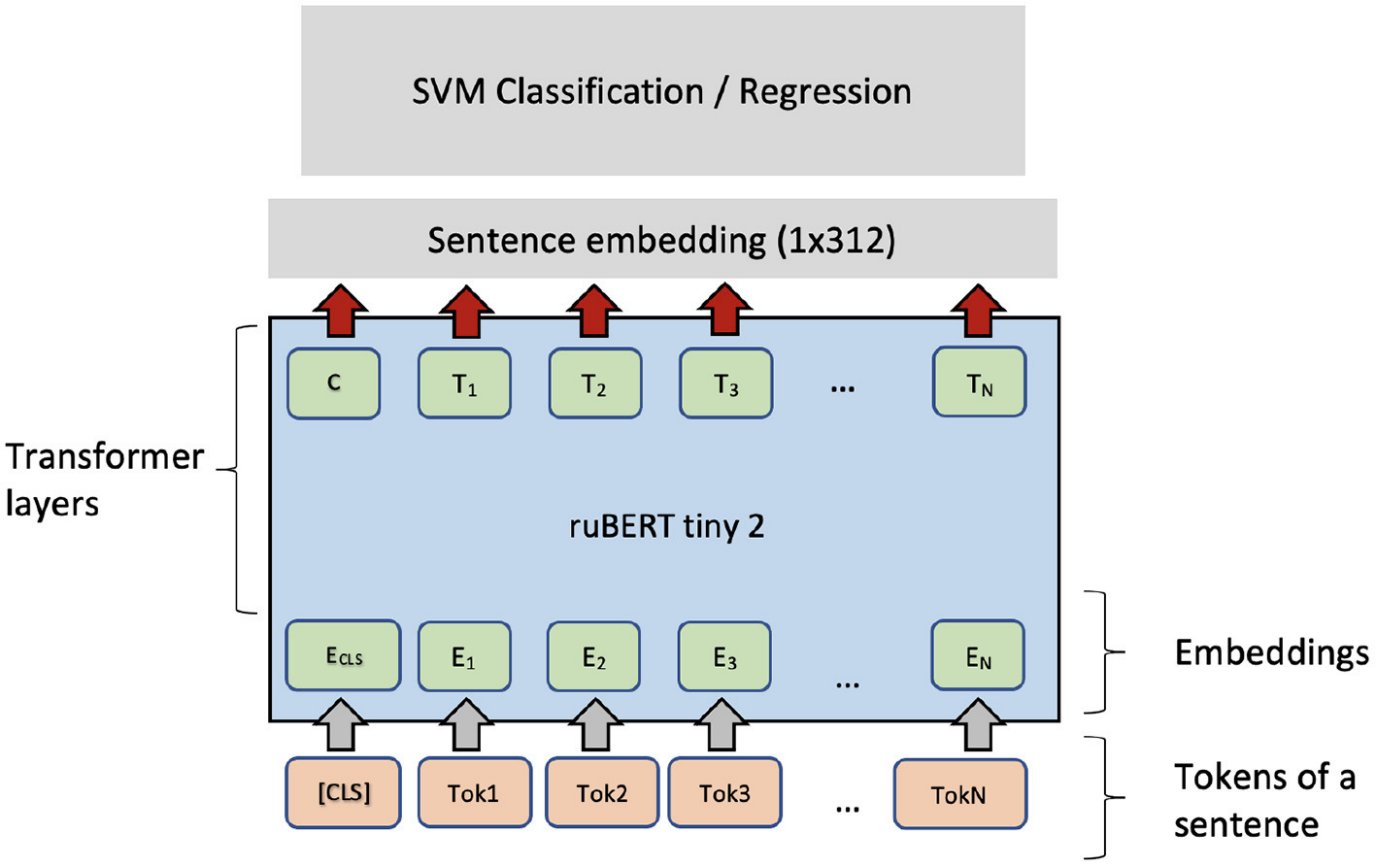
SVM + ruBERT-tiny2 architecture for single sentence complexity prediction.

**Figure 5:**
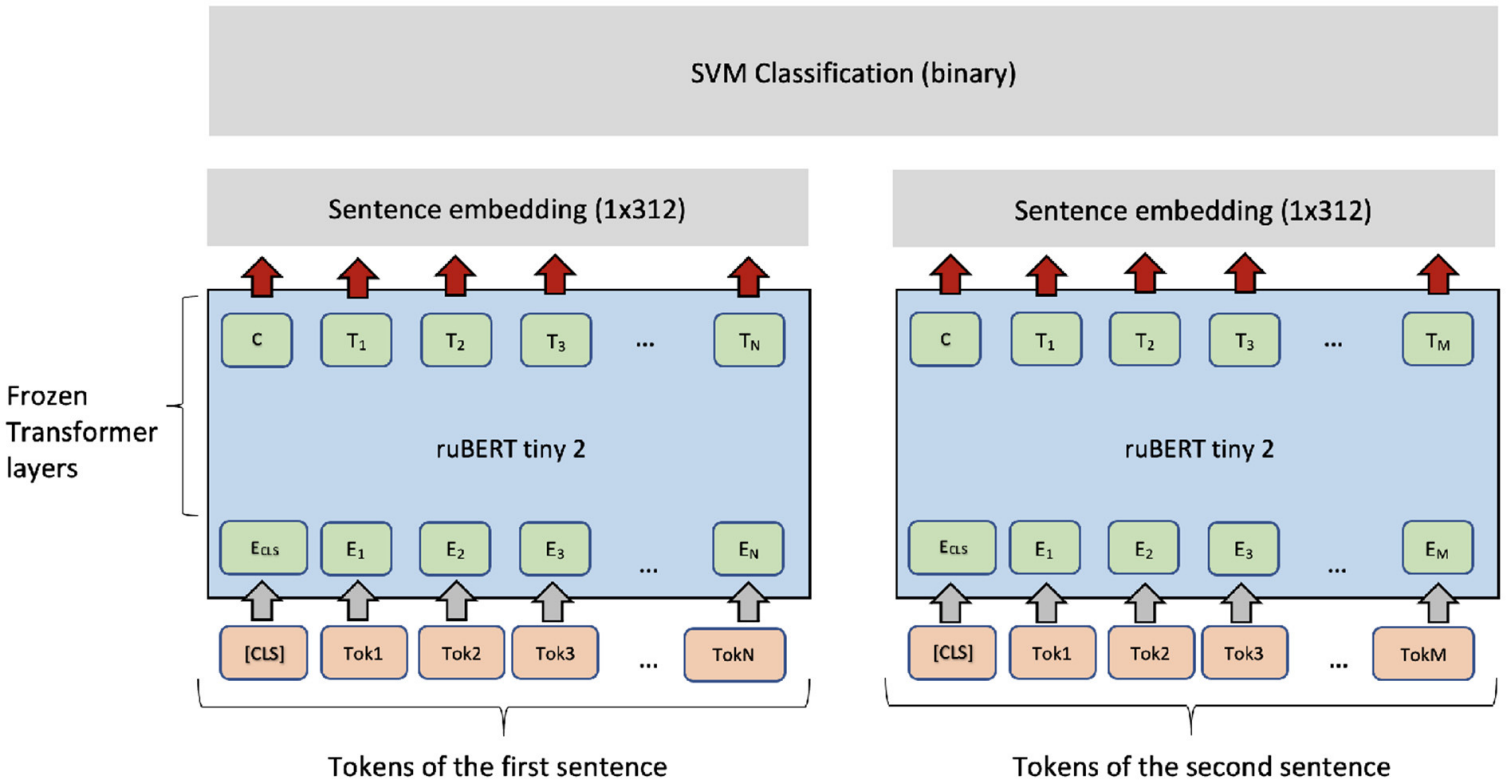
SVM + ruBERT-tiny2 architecture for classification and regression for sentence pairs.

Typically, fine-tuning a PLM needs optimizing many parameters in the transformer layers. Fine-tuning can be unstable and time-consuming. Therefore, another option is to use a PLM as a feature extractor. For each sentence, the pre-trained ruBERT-tiny2 sentence encoder (Dale, 2021) produces a fixed-length vector with 312 dimensions. The embeddings can be used as features for a high-level classifier (the SVM classifier). As Figure 4 shows, the same architecture can be applied in the regression setting if the SVM classifier is exchanged with an SVM regressor (SVR). For binary classification of sentence pairs, a similar architecture can be applied (see Figure 5).

The Transformer-based architectures process text input as a sequence of tokens. For many applications, this way of treating texts is natural and usually performs well. However, for text complexity analysis, syntactical features also proved to be useful, as mentioned by Solovyev et al. (2018b) and Iavarone et al. (2021). The sentence syntax tree does not form a sequence and, therefore, cannot be processed with a PLM. The Section 3.2.3 below presents an approach that combines syntactic and lexical information about a sentence and applies a graph neural architecture to predict sentence complexity.

#### 3.2.3. GNN model with fastText word embeddings

Graph neural network is a type of neural architecture intended to process data represented as graphs, e.g., for prediction properties of chemical molecules, syntactic trees, etc.; for more information, please, refer to the review of GNNs by Wu et al. (2020). Typically, GNNs are based on a message-passing mechanism when each node in a graph has an internal state (node embedding) that can be transferred to its neighbors. During message-passing, the node “sends” its state to adjacent nodes (neighbors). Thus, after each message-passing, all neighbors aggregate the messages from adjacent nodes. Usually, these messages are passed over the network for a fixed number of iterations. These message-passing steps are treated as network layers. Node embeddings aggregated after the whole process can be viewed as contextual representations. The context size depends on the number of layers.

This paper employed a Multi-layer Graph Convolutional Network (GCN) with self-attention^3^. The GCN is a model that has the following layer propagation rule to calculate the hidden representation of the *i*-th node $h_{i}^{\left(l+1\right)}$ using hidden representations of all its neighbors $h_{j}^{\left(l\right)}$ from the previous layer (*l*).


(1) $$\begin{array}{l} h_{i}^{\left(l+1\right)}=\sigma \left(\underset{j\in N\left(i\right)}{\sum }\frac{1}{c_{ij}}h_{j}^{\left(l\right)}W^{\left(l\right)}\right) \end{array}$$

Here, N(i) represents a set of neighbors of the *i*-th node. The ReLU activation function is used as a default setting for the non-linear transformation function σ(·). The matrix *W*^(*l*)^ contains trainable parameters of a convolution filter and the *c*_*ij*_ represents a normalization constant. At the last layer, each node of a graph has its vector representation; these representations are averaged and passed through a fully connected layer to train the model for classification or regression.

This paper uses the following GCN architecture. The first and the second layers apply the multi-head attention with six heads, and the third layer is a convolutional layer that outputs 64-dimensional vector for each node in a graph. The result is projected via a linear layer to the desired output, i.e., 11 or 3 values in case of multi-class classification, and a scalar value in case of regression.

**Figure 6:**
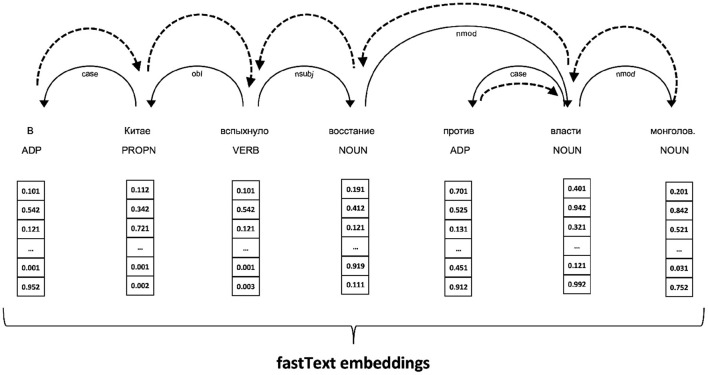
Constructing a graph input for GNN from a dependency tree (solid lines) augmented with backward edges (shown as dashed arcs). Each node in the graph is represented with a fastText embedding of the corresponding word.

In a sentence-level complexity prediction, each sentence is treated as a graph derived from a dependency syntax tree of the sentence. Precisely, the graph edges are constructed from the edges of a dependency tree for a given sentence, plus backward edges that help message-passing. Each tree node represents a token from the source sentence. When the GNN is initialized, node representation in the input layer are loaded from the fastText embedding for Russian (fastText, Bojanowski et al., 2016). Therefore, the input to the GNN combines syntactic features at the edge level with lexical features at the node level. An example of an input sentence is presented in Figure 6, where one can see the original sequence, fastText embeddings, and dependency tree with additional backward links. The dependency trees for all sentences were produced by DeepPavlov dependency parser. The proposed approach to using a GNN in sentence complexity evaluation is novel to the best of the author's knowledge.

## 4. Results

The experiment results are grouped according to the tasks formulated in Section 3.1.5. Regression task on the 1-s dataset is trained on the grade level value, while training on the 2-s dataset is done on the grade difference between two sentences that constitute a pair. In addition to the models described in Section 3.2, we trained and evaluated two linear regression models. The first one is a linear model of two predictors: SL and AWL. The second linear regression is trained on the sentence embeddings generated for an input sentence by a pre-trained model. Results on the test set for all models are presented in Table 5. The values in the first row of the table are absent because they correspond to linear regression that uses SL and AWL features. However, both sentences in each pair of the 2-s dataset have the same input values of SL and AWL. Obviously, linear regression would not produce any meaningful results in this case. On the 1-s dataset linear regression model performs worse than all others giving the maximum of both MSE = 8.34 and MAE = 2.32. Comparing the second and the third lines of the table, one can see that the support vector regression model works better on both datasets than linear regression. Both models work with contextual features generating 312-dimensional sentence embeddings through the RuBERT-tiny2 that is most likely the source of performance boosting.

**Table 5:** Regression of single sentence complexity (1-s data) and pairs (2-s data).

	**1-s data**			**2-s data**		
**Model**	***R* ^2^**	**MSE**	**MAE**	***R* ^2^**	**MSE**	**MAE**
Linear Reg. on two parameters (SL+AWL)	0.13	8.34	2.32	–	–	–
Linear Reg. on sentence embeddings	0.65	3.39	1.37	0.73	10.55	2.56
SVR on sentence embeddings	0.71	2.79	1.11	0.77	8.96	2.28
Transformer (1 layer, 4 heads, dim. = 64)	0.66	3.32	1.22	–	–	–
Fine-tuned RuBERT	0.80	1.96	0.80	0.98	0.79	0.56
GNN	0.73	2.58	1.10	0.97	1.15	0.75

On the 2-s dataset the SVR is outperformed by RuBERT and GNN because fixed-size vectors fail to represent a distinction between two sentences. The contextual sentence embeddings useful in the regression task on the 1-s dataset do not show good results on the 2-s dataset. The difference between grade levels of sentences cannot be less than three due to the setup of the margin; thus, the MAE = 2.28 derived by the SVR model is far from an acceptable result.

As expected, comparing GNN and the fine-tuned RuBERT shows that a more complex model (RuBERT) has higher performance that is still close to the GNN performance. RuBERT has a few hundred million parameters, while the GNN-based model has less than one million trainable parameters. Both RuBERT and the GNN models take into account structural features but do it differently. RuBERT does it via self-attention by training an *N*×*N* matrix of attention weights where each token can pass a message to any other token in the sentence. Despite the GNN model uses a similar mechanism, this model leverages the dependency tree structure to pass messages between tokens. Finally, the pure Transformer model works well, but experiments show that increasing the model complexity (e.g., by adding more layer) leads to overfitting.

Table 6 presents the results of classification models trained and evaluated on the 1-s dataset. Again, fine-tuned RuBERT shows better results than the SVM and GNN models. As expected, predicting sentence complexity with three target categories (low/medium/high) is much easier than classifying with 11 classes. On both tasks, according to the F1-macro score, the SVM and GNN models show similar quality, but they have different precision and recall values. SVMs have higher values of recall than precision, while GNN demonstrates the opposite behavior. This fact can be used when building ensemble models.

**Table 6:** Classification of sentences with 3 and 11 complexity categories.

**1-s Dataset**	**3 Classes**				**11 Classes**			
**Model**	**Acc**.	**F1**	**P**	**R**	**Acc**.	**F1**	**P**	**R**
SVM on sentence embeddings	0.85	75.78	73.61	81.08	0.61	44.33	42.12	51.94
Transformer	0.81	68.32	68.01	75.42	0.55	41.31	44.21	40.15
Fine-tuned RuBERT	0.87	81.99	82.67	81.38	0.68	55.89	56.30	55.76
GNN	0.82	73.48	75.53	71.95	0.62	48.04	50.98	46.99

Table 7 shows the results of the binary classification task on the 2-s dataset. Here, all three models perform quite well. The SVM performance is higher than it was before (see Table 2) which is due to the larger training dataset. The binary classification task can be considered as easy when the minimum margin is three grade levels.

**Table 7:** Binary complexity classification of sentence pairs.

**2-s Dataset (with margin** = **3)**				
**Model**	**Accuracy**	**F1**	**P**	**R**
SVM on sentence embeddings	0.93	93.18	93.18	93.18
Fine-tuned RuBERT	0.98	98.47	98.44	98.50
GNN	0.97	96.60	96.60	96.60

## 5. Analysis and discussion

Section 5 compares this study to prior research in terms of key findings and novelty. In addition, this section discusses limitations and directions for future work.

### 5.1. Novelty and application

In this study, a 2-s dataset contains pairs of sentences sampled from different books. Each sentence can describe completely different context that complicates the regression and classification tasks and forces a model to consider features not related to paraphrasing as it is usually done in related work (Inui and Yamamoto, 2001). Many researchers study how lexical and morphosyntactic features affect the text complexity of a sentence. The present paper proposes a new model that can be used to extract features for the SCE task. Indeed, the GNN operates directly with representations of tokens and syntax dependencies of a sentence. The last linear layer of the classifier aggregates the representation of each node with a weighted combination of individual node embeddings. This aggregation leads to a straightforward interpretation of the GNN model results. In fact, activations in the last linear layer can be interpreted as positions of words that directly affect model output.

**Figure 7:**
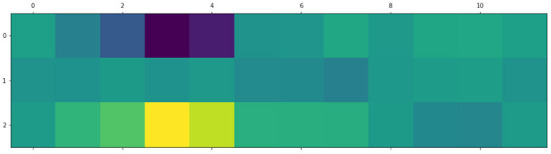
An activation pattern of the GNN for the input presented above in text. Columns correspond to graph nodes, rows correspond to three complexity classes. The maximum activation corresponds to words “mass professions” that “fire” for the label with the highest complexity.

For example, Figure 7 below shows an activation pattern for a sentence with the “high” complexity label. The input sentence is the following: Одной из массовых профессий в нынешнем столетии становится профессия программиста./“”. One of the mass professions in this century is the profession of a programmer. The mapping between nodes identifiers and sentence tokens the words is the following: (0: “ROOT,” 1: “Одной”/One, 2: “из”/of, 3: “массовых”/mass, 4: “профессий”/professions, 5: “в”/in, 6: “нынешнем”/this, 7: “столетии”/century, 8: “становится”/is, 9: “профессия”/profession, 10: “программиста”/programmer, 11: “.”). Simple sentences demonstrate an opposite pattern with high activations for “simple” words. Such property of the GNN can be transferred to edges and edge types of the dependency tree allowing interpretation of syntax dependencies from the point of text complexity, but it needs additional investigation. What is more, the analysis of the token-level activation can be applied in the lexical complexity prediction task. Note, that it is much harder to interpret the output of the BERT-based and the SVM-based models in the same manner. For the BERT-based model, the “[CLS]” token has a fixed embedding size of 768, while the SVM-based model produces 312-dimensional embeddings of input sentences.

Note, that in contrast to the work of Brunato et al. (2018) the present paper eliminates the effects of SL and AWL completely. The methods proposed in the paper need no sophisticated feature engineering and can be easily transferred to Italian and English datasets to compare classification and regression performance.

One can compare the above to the results that also show that SVM performs worse than the deep neural networks even when trained on contextual sentence embeddings. The best F1-scores reported in Lo Bosco et al. (2021) (for a binary classification) are around 0.88–0.89. Currently, the values of performance metrics are similar, but direct comparison is possible only on the same datasets.

### 5.2. Key findings

The study tests several neural architectures for the sentence-level complexity prediction task. The results show that fine-tuning of PLMs performs slightly better than training a GNN. The contextualized sentence embeddings are considered as an appropriate representation of the input text for the complexity prediction task. However, fixed sentence embeddings seem to be not enough for predicting the difference in the complexity levels between two sentences. The key difference with previous studies on SCE is that SL and AWL are not used as features. Finally, a proposed approach to using the GNN is promising for its performance and interpretability.

### 5.3. Future work

The future work of the study is organized in two directions. First, the plan implies multilingual and cross-lingual sentence complexity evaluation. In this direction, evaluation of models on Italian and English data, as well as training a multi-lingual model (either using a multilingual BERT or using multilingual embeddings in a GNN) can be done. The second direction is related to extending the GNN-based model taking into account the types of syntactic relations applying a relational convolutional graph network, and studying the usefulness of embeddings that the model is capable to produce.

## Data availability statement

The datasets and source code for this study can be found here: https://disk.yandex.ru/d/SMTIGk8GLx64EA.

## Author contributions

VI: conception and design of the study, formulation of research goals and aims, literature review, implementation, experiments, and writing the paper.

## Footnotes

^1^The classifier is described in detail in section 3.2.

^2^Experiments with different proportions (80/20) have shown similar results with some drop in performance.

^3^The implementation of the GCN architecture can be found in the Deep Graph Library (Wang et al., 2019).
